# Complete plastid genome sequence of *Erigeron breviscapus* (Asteraceae), an endemic traditional Chinese herbal medicine

**DOI:** 10.1080/23802359.2019.1605854

**Published:** 2019-11-18

**Authors:** Jing Meng, Linna Zhang, Zhiyuan Dong, Jun He

**Affiliations:** a College of Horticulture and Landscape, Yunnan Agricultural University, Kunming, China;; b Institute of Medicinal Plant, Yunnan Academy of Agricultural Sciences, Kunming, China;; c Kunming Institute of Botany, Chinese Academy of Sciences, Kunming, China

**Keywords:** *Erigeron breviscapus*, plastid genome, traditional Chinese herb, endemic species

## Abstract

*Erigeron breviscapus* is an important traditional Chinese herb endemic to China for cardiovascular and cerebral vessel diseases. Here, the complete plastid genome of *E. brevisapus* was sequenced using Illumina sequencing technology. The complete chloroplast genome size is 152,183 bp, including a large single-copy region of 84,705 bp, a small single-copy region of 18,112 bp and a pair of inverted repeat regions of 24,683 bp. The total GC content is 37.2%. The genome contains 113 genes, including 80 protein-coding genes, 29 tRNA genes, and four rRNA genes. Phylogenetic analysis showed that *E*. *breviscapus*, *Aster altaicus*, and *Eschenbachia blinii* formed a clade with 100% bootstrap support.

*Erigeron breviscapus* (Vaniot) Hand.-Mazz., also called ‘Duan Ting Fei Peng’ or ‘Deng Zhan Xi Xin’ in Chinese, is a perennial herb endemic to China, distributed in Yunnan, Sichuan, Guangxi, Guizhou, Xizang and Hunan provinces (Wu et al. 2011). *Erigeron breviscapus* is an important traditional Chinese herb, which has been listed in the updated version Chinese Pharmacopeia (China Pharmacopeia Committee 2015), and widely used for the treatment of stroke hemiplegia, retinal vein occlusion, promoting cerebral circulation, and reducing platelet destruction (Chen et al. 2018; Li et al. 1998). Scutellarin, the main chemical active component of *E. breviscapus*, is the natural specific drug for treatment of obdurate cerebrovascular disease and sequela of cerebral hemorrhage (Li et al. 2017). Unfortunately, due to overexploitation of wild resources, the wild populations of *E*. *breviscapus* have been severely destroyed (Yu and Chen 2002). To date, there is no report about the complete plastid genome of genus *Erigeron* including this important traditional Chinese medicine plant, *E*. *breviscapus*.

For the first time, we sequenced and characterized the complete plastid genome of *E*. *breviscapus*. The material was obtained from Gejiu’s Lianhua Mountain of Yunnan province (103°13′00.92″E, 23°20′06.14″N), and voucher specimen was deposited in the herbarium of KUN. Total genomic DNA was extracted using a modified CTAB method (Doyle and Doyle 1987). A paired-end library was constructed, and paired reads were then sequenced on Illumina Miseq platform (Illumina, San Diego, CA, USA). We assembled the reads through CLC Genomic Workbench v10 (CLC Bio., Aarhus, Denmark). All the contigs were checked against the reference genome of *Eschenbachia blinii* (KX 085421) using BLAST (https://blast.ncbi.nlm.nih.gov/). The web program DOGMA (http://dogma.ccbb.utexas.edu/) (Wyman et al. 2004) and Geneious v8.0.2 (Kearse et al. 2012) were utilized to annotate and construct a complete sequence, respectively. To determine the phylogenetic position of *E*. *breviscapus*, the whole chloroplast genome sequences of 13 species from Asteraceae were aligned by MAFFT v7 (Katoh et al. 2017), then a maximum likelihood (ML) phylogenetic tree was constructed using MEGA v 7.0 (Kumar et al. 2016).

The plastid genome of *E*. *breviscapus* is a circular DNA with 152,183 bp in length, and the genome sequence has been deposited in GenBank under accession number MK414770. Total genome contains a pair of inverted repeat (IRs) regions of 24,683 bp each seperated by a large single-copy (LSC) region of 84,705 bp and a small single-copy (SSC) region of 18,112 bp. The overall GC content of *E*. *breviscapus* plastid genome is 37.2%, and GC content (43.1%) in IRs regions is higher than those of LSC and SSC regions (35.0% and 31.0%, respectively). The complete chloroplast genome contains 113 genes, including 80 protein-coding (PCGs) genes, 29 transfer RNA (tRNA) genes, and four ribosomal RNA (rRNA) genes. Among them, 18 intron-containing genes were investigated: three of which (*ycf3*, *clpP*, and *rps12*) contained two introns, other 15 genes had one intron. Eight PCG genes, seven tRNA genes, and all four rRNA genes are duplicated in IR regions. The phylogenetic analysis indicated that *E*. *breviscapus* formed a monophyletic group with *Aster altaicus* and *Eschenbachia blinii*, with bootstrap support values of 100% (Figure 1).

**Figure 1.**
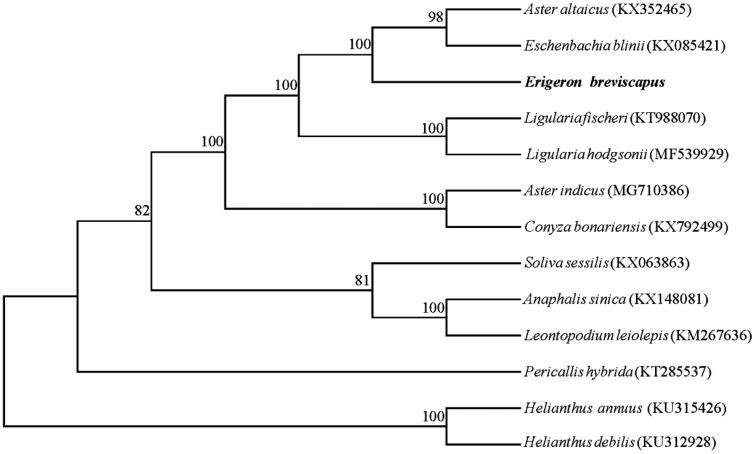
Maximum likelihood (ML) phylogenetic tree based on 13 complete plastid genome sequences. Bootstrap support values are shown on each node based on 1000 replicates.
